# Supplementary material for: Pocket MUSE: an affordable, versatile and high-performance fluorescence microscope using a smartphone
Source: Commun Biol. 2021 Mar 12;4:334. doi: 10.1038/s42003-021-01860-5 (PMC7955119; doi:10.1038/s42003-021-01860-5)
Supplement: Supplementary file 3 — Description of Additional Supplementary Files [file 42003_2021_1860_MOESM3_ESM.pdf]

Description of Additional Supplementary Files

File Name: Supplementary Video 1

Description: An example procedure for cheek cell cytology imaging using Pocket MUSE.

File Name: Supplementary Video 2

Description: An example video showing suspended bacteria stained with acridine orange under Pocket MUSE preview.

File Name: Supplementary Video 3

Description: A live roundworm moving around a piece of grimmia moss imaged with 3 different modalities using Pocket MUSE.
